# Agrobacterium-Mediated Transformation of Chrysanthemum with Artemisinin Biosynthesis Pathway Genes

**DOI:** 10.3390/plants9040537

**Published:** 2020-04-21

**Authors:** Aleksey Firsov, Tatiana Mitiouchkina, Lyubov Shaloiko, Alexander Pushin, Alexander Vainstein, Sergey Dolgov

**Affiliations:** 1 Branch of the Shemyakin-Ovchinnikov Institute of Bioorganic Chemistry of the RAS, Moscow Region, 142290 Pushchino, Russia; tatiana@planta.bio (T.M.); shaloiko@yandex.ru (L.S.); aspushin@gmail.com (A.P.); dolgov@bibch.ru (S.D.); 2 The Hebrew University of Jerusalem, Robert H. Smith Faculty of Agriculture, Food and Environment, POB 12, Rehovot 76100, Israel; alexander.vainstein@mail.huji.ac.il

**Keywords:** transgenic plants, chrysanthemum, artemisinin, metabolic engineering

## Abstract

Artemisinin-based drugs are the most effective medicine for the malaria treatment. To date, the main method of artemisinin production is its extraction from wormwood plants *Artemisia annua* L. Due to the limitation of this source, considerable efforts are now directed to the development of methods for artemisinin production using heterologous expression systems. Artemisinin is a sesquiterpene lactone, synthesized through the cyclization of farnesyl diphosphate involved in other sesquiterpene biosynthetic systems. *Chrysanthemum* species as well as *A. annua*, belong to *Asteraceae* family, and had been characterized by containing highly content of sesquiterpenes and their precursors. This makes chrysanthemum a promising target for the production of artemisinin in heterologous host plants. Chrysanthemum (*C. morifolium* Ramat.) was transformed by *Agrobacterium tumefaciens* carrying with the binary vectors p1240 and p1250, bearing artemisinin biosynthesis genes coding: amorpha-4,11-diene synthase, artemisinic aldehyde Δ11(13) reductase, amorpha-4,11-diene monooxygenase (p1240 was targeted to the mitochondria and p1250 was targeted to the cytosol), cytochrome P450 reductase from *A. annua*, as well as yeast truncated 3-hydroxy-3-methylglutarylcoenzyme A reductase. This study obtained 8 kanamycin-resistant lines after transformation with the p1240 and 2 lines from p1250. All target genes were detected in 2 and 1 transgenic lines of the 2 vectors. The transformation frequency of all target genes were 0.33% and 0.17% for p1240 and p1250, relative to the total transformed explant numbers. RT-PCR analysis revealed the transcription of all transferred genes in two lines obtained after transformation with the p1240 vector, confirming the possibility of transferring genetic modules encoding entire biochemical pathways into the chrysanthemum genome. This holds promise for the development of a chrysanthemum-based expression system to produce non-protein substances, such as artemisinin.

## 1. Introduction

Currently, the most effective drugs for the treatment of malaria are drugs combined with artemisinin, the use of such drugs is recommended by WHO resolution (WHA60.18, May 2007). Additionally, artemisinin and its derivatives are also effective to a number of viruses, many lines of human tumor cells, some tropical parasitic diseases and a number of livestock diseases [1]. Artemisinin is a terpenic lactone found in wormwood plant (*Artemisia annua* L.). The content of artemisinin in *A. annua* is usually in the range of 0.01–0.8% of dry weight, which is not enough to meet the global demand for artemisinin-containing drugs [2,3]. Improvement of cultivation techniques and breeding of *A. annua* has not led to a significant improvement in the yield of artemisinin [4,5]. In this connection, the possibility of producing artemisinin by methods alternative to extraction from natural sources is currently being actively studied. The biochemical pathway of artemisinin synthesis has now been comprehensively studied [6,7] and this allowed advancing research on artemisinin production in heterologous systems.

For example, Farhi et al. (2011) transferred a genetic module from 5 genes of artemisinin synthesis pathway by agrobacterium-mediated method to the tobacco plants [8]. The accumulation of artemisinin in tobacco was 0.0068 mg/g dry leaf weight (DW). In stable transformed moss of *Physcomitrella patens* artemisinin accumulation reached to 0.2 mg/g DW [7]. In experiments on transient expression of artemisinin biosynthesis genes in *Nicotiana benthamiana* plants, artemisinin accumulated at a level of 0.003 mg/g DW [9]. A highest level (0.8 mg/g DW of leaf tissues) of artemisinin accumulation was observed in double transformed tobacco plants, the artemisinin pathway genes were transferred into the genome and the mevalonate pathway genes (MVA pathway) were transferred in the plastome [10]. These studies confirmed the fundamental possibility of artemisinin production in heterologous plants.

**Figure 1:**
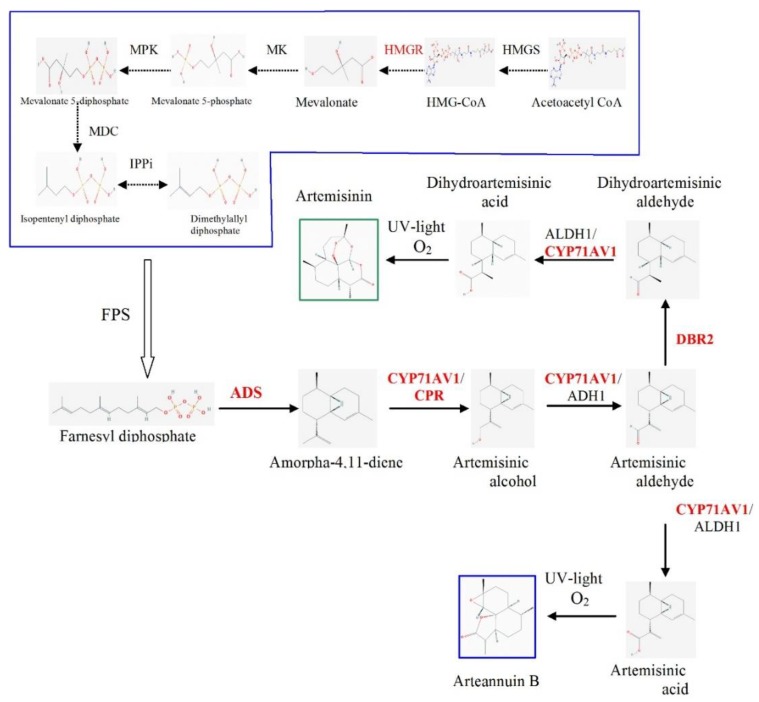
Biosynthetic pathway of artemisinin. The mevalonic acid (MVA) pathway is shown in a blue frame. Artemisinin pathway: ADS: amorpha-4,11-diene synthase; CYP71AV1: amorpha-4,11-diene monooxygenase; CPR: cytochrome P450 reductase; ADH1: alcohol dehydrogenase; DBR2: aldehyde Δ11(13) reductase; ALDH1: aldehyde dehydrogenase. MVA pathway: HMGS: 3-hydroxy-3-methylglutaryl-CoA synthase; HMGR: 3-hydroxy-3-methylglutaryl-CoA reductase; MK: mevalonate kinase; MPK: mevalonate 5-phosphate kinase; MDC: mevalonate 5-diphosphate decarboxylase; IPPi: isopentenyl diphosphate isomerase; FPS, farnesyl diphosphate synthase. Artemisinin biosynthesis genes cloned in p1240 and p1250 vectors are shown in red.

Artemisinin is synthesized from two isoprenoid precursors, isopentenyl diphosphate (IPP) and dimethylallyl diphosphate (DMAPP). IPP and DMAPP are condensed by farnesyl diphosphate synthase (FPPS/FPS) into farnesyl diphosphate (FPP, farnesyl pyrophosphate), the C15 sesquiterpenoid precursor [6,11]. FPP is converted by amorpha-4,11-diene synthase (ADS) to amorpha-4,11-diene (AD) and further into artemisinin (Figure 1). The limited pool of IPP and DMAPP will restrict FPP production and consequently that of artemisinin. This assumption is supported by a study of Malhotra et al. (2016) [10], when the transfer of the mevalonate pathway genes into the plastome of tobacco led to a strong increase of artemisinin accumulation. In this study, a direct correlation was observed between the synthesis of IPP and DMAPP as well the artemisinin production.

An obvious way to increase artemisinin production in heterologous systems is to select a plant-host with a naturally high content of terpenoids and their precursors. Many such plants are known, especially among crop plant belonging to flavor herbs or medicines, such as chrysanthemum [12,13,14]. The high content of various terpenoids is characteristic of the widespread *Chrysanthemum morifolium* Ramat, which is one of the leading ornamental crops, second to roses, cultivated almost everywhere [15,16]. Noteworthy, the methods of biotechnology and genetic engineering of chrysanthemum have already been developed in detail. The cultivation technologies of chrysanthemum also have been elaborate both for field and for greenhouses conditions [17]. To date, whole-genome sequencing of the diploid species of *C. seticuspe* and a transcriptome analysis of *C. morifolium* have been carried out [18,19]. The data obtained in these studies can promote a more in-depth understanding of biochemical processes in chrysanthemum, especially in metabolic engineering. Thus, chrysanthemum may be a promising platform for artemisinin production *in planta* as a heterologous host.

The aim of this work was to explore the feasibility of agrobacterium-mediated transformation of chrysanthemum with artemisinin biosynthesis genes. Here, we report the successful transformation of chrysanthemum with five genes of artemisinin biosynthesis pathway, i.e., genes responsible for production of dihydroartemisinic acid and mevalonate and show their transcription in the transgenic plants.

## 2. Results and Discussion

### 2.1. Agrobacterium-Mediated Transformation of Chrysanthemum Plants with p1240 and p1250 Vectors

**Figure 2:**
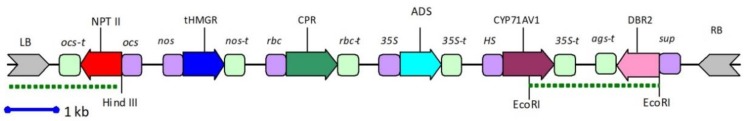
Schematic depiction of the expression cassettes of vectors p1240 and p1250 to artemisinin production in chrysanthemum. tHMGR: 3-hydroxy-3-methylglutaryl-coenzyme A reductase, CPR: cytochrome P450 reductase, ADS (mtADS): amorpha-4,11-diene synthase, CYP71AV1: amorpha-4,11-diene monooxygenase, DBR2: artemisinic aldehyde Δ11(13) reductase. Purple boxes indicate promoters, pale green boxes: terminators; ocs: octopine synthase; nos: nopaline synthase; rbc: rubisco; 35S: cauliflower mosaic virus (CaMV) 35S; HS: hps18.1 promoter; sup: superpromoter; ags: agrocinopine synthase. LB and RB: left and right T-DNA borders, respectively. EcoRI and Hind III: sites used for Southern blot analysis. Dotted lines shows probe-specific parts of T-DNA.

Two variants of the vectors were used in our study—with localization of amorpha-4,11-diene synthase in the cell cytoplasm (p1250)—or in mitochondria (p1240). It was previously reported that switching subcellular localization of introduced ADS to mitochondria leads to a strong increasing of amorpha-4,11-diene accumulation, the artemisinin precursor [20,21,22]. Studying the influence of artemisinin biosynthesis enzymes localization in various cell compartments on its accumulation is important for the obtaining of plants producing recombinant artemisinin. Vectors p1240 and p1250 were identical and differed only in the presence of a mitochondrial targeting signal sequence in the ADS gene (vector p1240) or its absence (vector p1250) (Figure 2).

**Figure 3:**
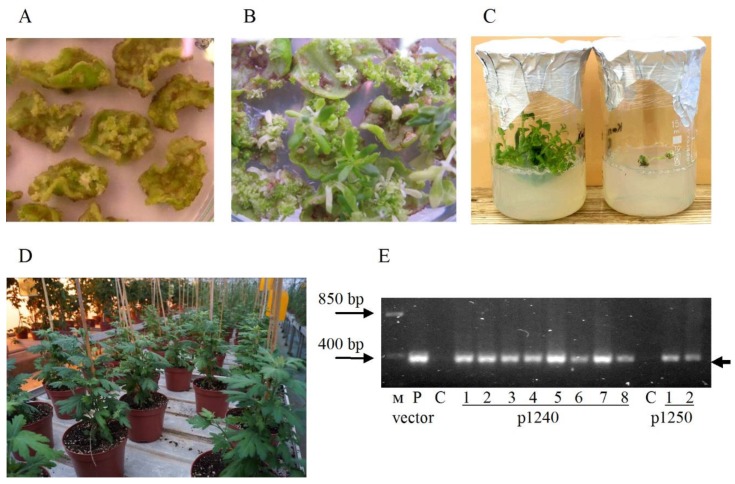
*Agrobacterium*-mediated transformation of chrysanthemum. (**A**) Callus induction on the leaf explants. (**B**) Adventive shoots regeneration on the medium with 35-mg/L kanamycin. (**C**) Chrysanthemum transformants on the multiplication medium with kanamycin: on the left is a kanamycin-resistant plant and on the right is sensitive to it. (**D**) Transgenic chrysanthemum plants in a greenhouse. (**E**) PCR analysis of NPT II gene in kanamycin-resistant chrysanthemum plants and the expected length of the amplified fragment was 381 bp (arrow indicated). C: nontransformed control plant; P: DNA of p1240. Numbers denote independent chrysanthemum lines. M: molecular size marker.

Chrysanthemum leaf explants were transformed with the p1240 and p1250 vectors. The mass induction of kanamycin-resistant calli was observed after three weeks of explants cultivation on a regeneration medium with 35.0-mg/L kanamycin (Figure 3A). The calli vigorously grew on a kanamycin-containing medium. The shoot regeneration began after two months of cultivation; the adventitious shoots arose from calli (Figure 3B). On average, 1–2 adventive shoots were formed per one regenerating explant. The green and normally growing regenerants reached 7–10 mm were cut off from the explants and further cultivated on a multiplication medium containing 35-mg/L kanamycin for further selection and proliferation. At this stage, about 80% of the regenerants turned chlorotic and died, the rest grew and multiplied normally (Figure 3C). When the shoots of kanamycin-resistant shoots reached a height of 3–4 cm, they were planted in the rooting medium containing 35.0-mg/L kanamycin. After 3 weeks cultivation, the rooted plants with no signs of toxic effect from kanamycin were adapted to the conditions in vivo and then cultivated in a greenhouse (Figure 3D).

At this stage, the kanamycin-resistant chrysanthemum plants were analyzed using PCR to determine the presence of the NPT II gene. A DNA fragment of the expected size was amplified from the DNA samples of all studied kanamycin-resistant lines (Figure 3E). In DNA samples from non-transformed plants, amplification of the NPT II fragment was not observed.

The transgenic chrysanthemum did not differ morphologically from the non-transformed counterparts. The development and growth rate of these plants in the greenhouse did not differ from the corresponding characteristics of the non-transformed plants. In total, ten independent transgenic lines of chrysanthemum were obtained, 8 lines of 1240 and 2 of 1250.

### 2.2. Analysis of the Integration of Artemisinin Biosynthetic Pathway Genes in Transformed Plants

**Figure 4:**
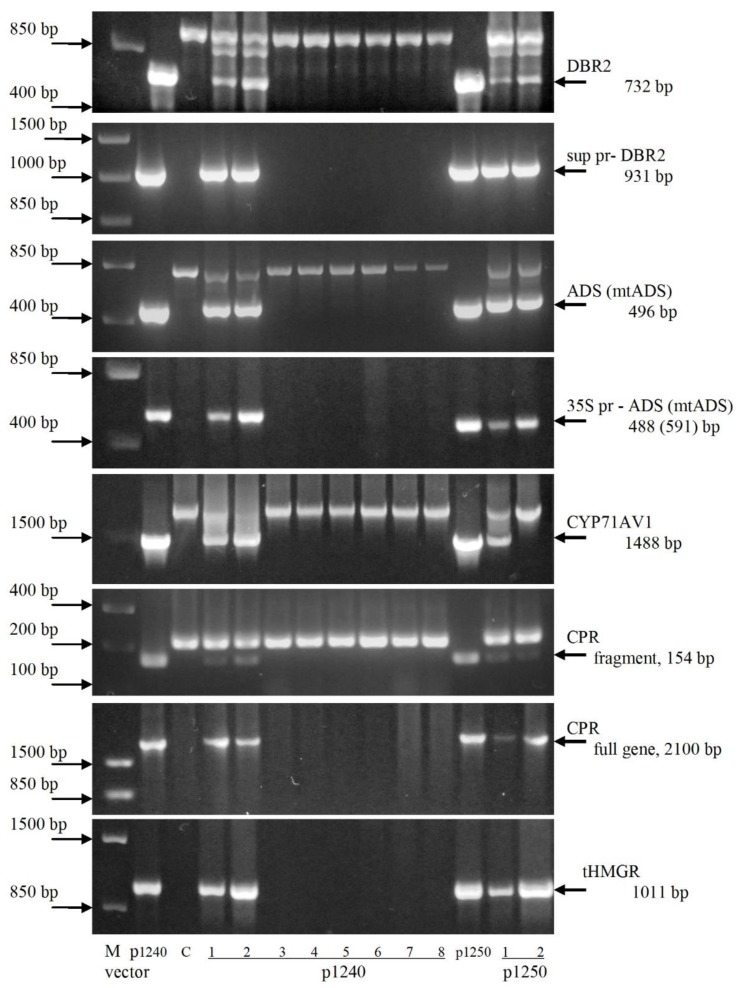
PCR analysis of transgenic chrysanthemum lines. DBR2: artemisinic aldehyde Δ11(13) reductase; CYP71AV1: amorpha-4,11-diene monooxygenase; ADS (mtADS): amorpha-4,11-diene synthase; CPR: cytochrome P450 reductase; tHMGR: 3-hydroxy-3-methylglutaryl-coenzyme A reductase. C: nontransformed plant, negative control; p1240 and p1250: DNA of plasmid p1240 and p1250, respectively, positive control. Numbers denote independent transgenic lines. The arrow marks the target fragments. The expected amplified fragments lengths are indicated by bp. M: molecular size marker.

The presence of artemisinin biosynthetic pathway genes in the transgenic chrysanthemum plants were studied using PCR with primers specific to the DBR2, ADS, mtADS, CYP71AV1, tHMGR and CPR genes, respectively. All the target genes were detected in two (1240/1 and 1240/2) out of eight lines, obtained after transformation with vector p1240, and none of these genes were detected in the rest six transgenic lines (Figure 4). All five artemisinin biosynthesis genes were detected in line 1250/1 and the CYP71AV1 gene was not revealed in 1250/2. In DNA samples from non-transformed plants, the fragments of the expected length were not amplified. Taken all together, we obtained 3 lines of chrysanthemum plants transformed with all five target genes.

In addition to PCR products with expected sizes, at using of gene-specific primers for the DBR2, ADS, CYP71AV1 and CPR genes the larger fragments were observed in all DNA samples, including those from non-transformed plants (Figure 4). We hypothesized that this is the result of amplification of chrysanthemum genome sequences, resembling the corresponding sequences from *A. annua*. Genome sequence analysis of the model species of *Chrysanthemum seticuspe* confirms the presence of such sequences in chrysanthemum [19]. When used primer pairs in which the forward primer was annealed to the heterologous promoter sequence, the target DBR2 and ADS genes were clearly detectable. Presence of the CPR gene was confirmed using a primer pair amplifying its full-length sequence. The tHMGR gene from yeast was amplified only in transgenic plants.

**Figure 5:**
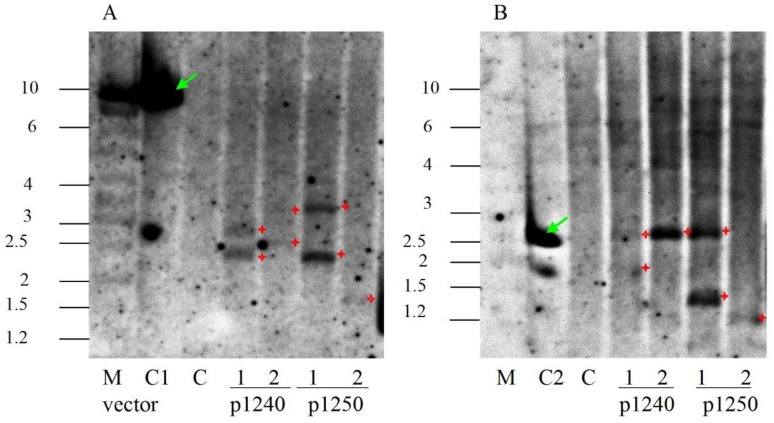
Southern blot analysis of transgenic chrysanthemum lines. (**A**) The hybridization result of probe to NPT II gene followed by digesting the DNA with Hind III; (**B**) The hybridization result of probe to DBR2 gene followed by digesting the DNA with EcoRI. C1: DNA of plasmid p1240, positive control; C2: DNA of plasmid p1250, positive control; C: nontransformed plant, negative control. Numbers denote independent transgenic lines. M: molecular size marker, kb. Stars mark positive signals after hybridization with probe. The arrow indicates the signals after hybridization with positive controls.

To further confirm the transgenic origin of the obtained lines, Southern blot analysis (SB) was performed and results matched the results of PCR. Genomic DNA were digested with Hind III followed by SB hybridization, which is expected to yield a DNA fragment longer than 1.7 kb in length in transgenic plants allowing to estimate the copy number of the T-DNA insertions. Southern blot analysis confirmed the presence of NPT II in the genomes in 4 transgenic lines and the lengths of the detected fragments corresponded with expected (Figure 5A). The NPT II gene in lines 1240/1, 1240/2 and 1250/1 was presented in two copies, in line 1250/2 was single copy.

In the case of restriction with EcoRI, a 2.4 kb fragment that includes the T-DNA sequence between the EcoRI sites of the DBR2 and CYP71AV1 genes should be revealed (Figure 2). Hybridization of the digested genomic DNA using a probe to DBR2 showed the presence of expected length fragments in the lines 1240/1, 1240/2 and 1250/1 (Figure 5B). In the lines 1240/1 and 1250/1, shorter fragments with a length of 1.2–1.8 kb were also detected. In the 1250/2 line, the fragment was detected corresponding to a size of 1.3 kb, i.e., significantly lower than expected. This may be due to a deletion of the CYP71AV1 gene sequence, which is consistent with the PCR analysis of this line.

SB analysis results confirmed the integration of vector T-DNA into the genome of studied lines. In lines 1240/1, 1240/2 and 1250/1, the T-DNA insertion is presented in two copies and in line 1250/2 is in one copy that are typical for agrobacterium-mediated transformation. Hybridization with a probe to the DBR2 gene showed the presence of a fragment of the expected length in the lines 1240/1, 1240/2 and 1250/1. In the 1240/2 line, the target fragment was detected as a single band, which indicates the correct transfer of two copies of T-DNA. In lines 1240/1 and 1250/1, SB revealed the presence of additional bands with a molecular weight lower than expected, which point to the incomplete transfer of one of T-DNA copies. The SB analysis also confirmed the PCR results of incomplete T-DNA in the 1250/2 line.

### 2.3. RT-PCR Analysis of Artemisinin Biosynthesis Genes Expression

**Figure 6:**
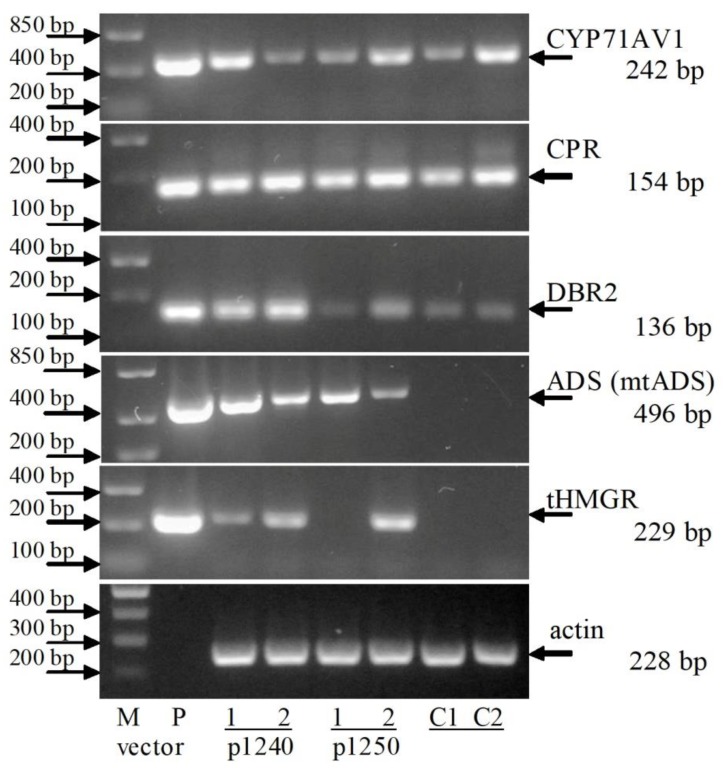
RT-PCR analysis of chrysanthemum lines. C1 and C2: nontransformed plants, negative controls; P: DNA of plasmid p1240, positive control. Actin: actin of *C. morifolium*, RT-PCR control. Numbers denote independent transgenic lines. M: molecular size marker. The expected length of the amplified fragment are indicated by bp.

The expression of artemisinin biosynthesis genes at the transcription level in lines 1240/1, 1240/2, 1250/1 and 1250/2 were studied using RT-PCR (Figure 6). Fragments corresponding to cDNA sequence of ADS gene were amplified in all transgenic lines. Amplification of tHMGR cDNA fragments was also observed in transgenic plants except for line 1250/1; in this line, the corresponding fragment was not amplified. This is probably due to the gene sequence damage during the T-DNA transfer into the plant genome. In non-transformed plants, amplification of ADS and tHMGR cDNA was not observed.

Using primer pairs corresponding to CYP71AV1, CPR and DBR2, the cDNA fragments were amplified both in transgenic lines and in samples obtained from non-transformed plants. This is consistent with the results of PCR, when using gene-specific primers amplification of the corresponding fragments with genome DNA was observed in samples obtained from both transgenic and control plants (Figure 4). The overall results of chrysanthemum transformation experiments and RT-PCR analysis of target genes transcription are shown in Table 1.

### 2.4. Artemisinin Assay in the Transgenic Plants

A TLC analysis was performed to preliminary evaluate the artemisinin accumulation. The lines 1240/1 and 1240/2 were chosen for the study, in these lines the transcription of all target genes was confirmed. Line 1250/1, in which the transcription of the tHMGR gene was not detected; 1250/2, into which the CYP71AV1 gene was not transferred, were not analyzed.

**Figure 7:**
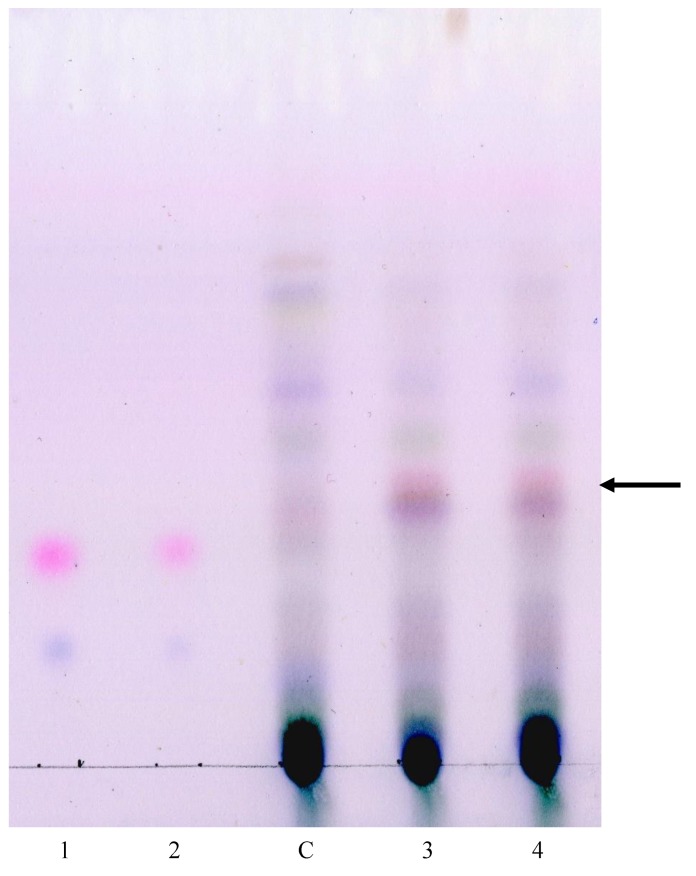
TLC analysis of artemisinin accumulation in transgenic chrysanthemum plants. Lanes 1 and 2: mixed standards of artemisinin and artemisinic acid (125 ng of each, lane 1; 62.5 ng of each, lane 2); C: non-transformed plant; 3: line 1240/1; 4: line 1240/2.

The results of TLC analysis are shown in Figure 7. In the studied lines, after development with anisaldehyde, a band was detected that was absent in non-transformed plants. The band had a pink color, typical of artemisinin. In addition, in this region of the chromatogram, a clearly visible unidentified blue band was observed, which was absent in untransformed plants. The band corresponding to artemisinic acid was not detected in the samples. Thus, the results of TLC analysis indicate a modification of plant metabolism after their transformation by artemisinin pathway genes.

As a result of the experiments, we obtained eight chrysanthemum lines after transformation with the p1240 vector and two lines after transformation with the p1250 vector. Based on the number of kanamycin-resistant lines, the transformation frequency was 1.3% and 0.3% of transformed explants. Of the eight transgenic lines obtained after transformation with the p1240, all target genes were detected in two lines and only one line containing all five target genes was obtained after transformation with the p1250. Thus, the frequency of chrysanthemum transformation of all target genes were between 0.33% and 0.17%. In most studies, the frequency of chrysanthemum transformation of varies in the range of 1–8% of transformed explants number [23,24,25]. The values are not high in this study. Since previous studies were transferred only 2–3 genes to the chrysanthemum genome, the chrysanthemum transformation by a large number of genes are unknown to us.

Currently, studies in the field of multiple gene transfer are carried out using model plants and easily transformed crops. For instance, 10 different genes as part of 28.5-kb T-DNA were possibly transferred into the *Arabidopsis thaliana* genome [26], 8 genes important for symbiosis with rhizobia (T-DNA size of 74 kb) were able to be transferred into *Medicago truncatula* [27], seven carotenoid biosynthesis genes in a single 17 kb T-DNA were able to be transferred into *Brassica napus* [28] and 32-kb T-DNA with ten insect resistance genes were successfully introduced into the elite line of maize and their expression in plants was shown [29]. This study was the first that introduced six genes simultaneously into the chrysanthemum genome with a size of about 14.0 kb T-DNA.

It should be noted that even small differences in the nucleotide sequence of T-DNA had a significant impact on the efficiency of its transfer during transformation. During transformation with the p1240 vector, only in two lines did the transfer of target genes take place; in the other 6 lines, none of them was detected. At the same time, during transformation with the p1250 vector, which differed from p1240 only in the absence of a signal peptide sequence in the ADS gene, the transfer of target genes was observed in both lines obtained.

RT-PCR analysis confirmed the transcription of heterologous ADS and tHMGR genes in transgenic plants. At the same time, using gene-specific primers for CYP71AV1, CPR and DBR2, amplification of cDNA fragments was observed both in transgenic lines and in non-transformed plants. TLC analysis revealed bands in the studied transgenic lines that were absent in the control plants, including those with a pink color typical of artemisinin. These data indicate a modification in the metabolism of transgenic plants under the influence of artemisinin pathway genes. Evaluation the recombinant artemisinin accumulation in the transgenic plants and a more thorough analysis of various genes influence on the artemisinin biosynthesis will be the next step of our study.

For metabolic engineering, it may be more applicable to transfer the biosynthesis module genes into plants not as one large T-DNA, but as a part of several smaller T-DNAs. Such a transfer can be carried out, in particular, by the method of co-transformation, when the explants are transformed simultaneously with several vectors. Other approach is the transformation of previously obtained and characterized lines carrying biosynthetic module genes (or part of them) with vectors with others studied genes (double transformation). It is also possible to obtaining a set of transgenic lines, each of which carries a part of the genetic module, with the subsequent crossing between them. These methods allow greater research flexibility by opportunities of combining various biosynthesis genes of the target product or its precursors. In addition, the last two approaches allow the use in studies the lines with the maximum expression of heterologous genes, thereby minimizing T-DNA position effect. The effectiveness of these approaches has been repeatedly confirmed; they will be used in our further research.

Thus, the data obtained in this study show the possibility of transferring gene groups encoding whole biochemical modules into the chrysanthemum genome. This opens up new routes for the development of chrysanthemum plant-based expression systems for the production of non-protein substances, including artemisinin.

## 3. Materials and Methods

### 3.1. The Plant Transformation Vectors

The chrysanthemum plants were transformed with p1240 and p1250 vectors. The vectors were constructed by cloning the genes of the artemisinin biosynthesis pathway into the pRCS16F vector using intermediate vectors of the pSAT series [8,30]. The following genes of *Artemisia. annua* were cloned into the pRCS16F vector: amorpha-4,11-diene synthase (ADS), artemisinic aldehyde Δ11(13) reductase (DBR2) and amorpha-4,11-diene monooxygenase (CYP71AV1). CYP71AV1 catalyzes amorpha-4,11-diene to form artemisinic aldehyde via artemisinic alcohol as well as conversion dihydroartemisinic aldehyde into dihydroartemisinic acid (Figure 1). Cytochrome P450 reductase (CPR) from *A. annua* was cloned into vector to prevent the accumulation of inactive oxidized P450. The vectors also contained a truncated and deregulated 3-hydroxy-3-methylglutarylcoenzyme A reductase (tHMGR) from yeast to increase the supply of precursor from the mevalonate (MVA) pathway for artemisinin production.

The amorpha-4,11-diene synthase gene was cloned in two variants—without a signal sequence (localization of ADS in the cytosol, vector p1250) and with the signal sequence of сytochrome C oxidase subunit 4 (COX4) from *Saccharomyces cerevisiae* (mtADS, localization in mitochondria, vector p1240). Thus, the engineered vectors differed from each other solely in the absence (p1250) or presence (p1240) of the COX4 signal peptide nucleotide sequence. The expression cassettes of vectors used in this study are presented in Figure 2.

The plasmids were transferred into *Agrobacterium tumefaciens* CBE21 and used for transformation. *A. tumefaciens* was grown overnight in liquid LB medium at 28 °C and 140 rpm. The bacterial cells were washed twice in liquid hormone-free Murashige and Skoog (MS) medium by centrifugation for 5 min (4000× *g*), and the bacterial pellet was resuspended in this medium to an OD600 of 0.2.

### 3.2. Agrobacterial Transformation of Chrysanthemum

The chrysanthemum plants (*Chrysanthemum morifolium* Ramat) of the cultivar White Snowdon were used in all experiments. Agrobacterium-mediated transformation and in vitro culture of chrysanthemum plants were performed as described previously [31,32].

Briefly, stock chrysanthemum plant were multiplicated on Murashige and Skoog medium (MS) with 1.0-mg/L 6-benzylaminopurine (BA), 0.1-mg/L indole-3-acetic acid, 0.5-mg/L gibberellic acid, 30 g/L sucrose and 0.8% agar. The shoot of 1.5–2.0 cm were rooted on the rooting medium (contained two times diluted MS salts, containing the macro- and micro-elements, vitamins MS, 10 g/L sucrose and 0.5-mg/L indolebutyric acid). The explants (the leaf pieces approximately 1 × 1 cm) were cut from three weeks old rooted plants. The leaf explants were incubated in bacterial suspension for 20 min, then placed upside down on regeneration medium (MS medium with 2.0-mg/L BA, 0.5-mg/L naphthaleneacetic acid) without antibiotics. After three days co-cultivation with agrobacterium, the explants were transferred to the regeneration medium (the regeneration medium containing of 500-mg/L cefotaxim and 35-mg/L kanamycin) and were subcultured every 3 weeks. The regenerants reaching 7–10 mm were cut off from the explants and further cultivated on a multiplication medium with 35.0-mg/L kanamycin and 500-mg/L cefotaxim. When the kanamycin-resistant shoots reached a height of 3 to 4 cm, they were transferred on the rooting medium containing 35.0-mg/L kanamycin and 250-mg/L cefotaxim. The rooted, vigorously growing kanamycin-resistant plants were than adapted to conditions in vivo and cultivated in a greenhouse.

### 3.3. PCR and Southern Blot Analysis

For PCR and Southern blot analysis, the genomic DNA of chrysanthemum was isolated from kanamycin-resistant and nontransformed control plants grown in greenhouse using the method of Dellaporta et al. [33]. DNA preparations were checked for suitability to PCR amplification using primers to actin of chrysanthemum (Figure S1A).

The plants were pre-tested for the absence of agrobacterial contamination using primers virBF and virBR that amplify the *virB* gene of *A. tumefaciens* (Table S1). The primer sequences used for detection of artemisinin biosynthesis genes in transgenic plants; the PCR regimes are presented in Table S1 of Supplementary Material.

Chrysanthemum genomic DNA (70 µg) was digested overnight at 37 °C with 100 U Hind III (cuts the T-DNA of p1240 and p1250 between the NPT II coding sequence and its octopine synthase promoter) or EcoRI (cuts out DNA fragment between DBR2 coding sequence and CYP71AV1) (Figure 2). Digestion by Hind III was used to evaluate the copy number of the T-DNA inserts, EcoRI allows to further confirm the integration of right border (RB) part of T-DNA into the plant genome. The DNA of non-transformed chrysanthemum plants digested with Hind III or EcoRI was used as a negative control.

After agarose gel (0.8%) electrophoresis, the digestion products were transferred and immobilized onto Hybond+ membrane (Amersham, USA) following the manufacturer’s instructions. The DNA probe was constructed by PCR using plasmid p1240 as the template and primer pairs NPT IIF–NPT IIR and DBR2F–DBR2R to amplify the sequences of NPT II and DBR2 genes. DNA probes (381 bp of NPT II and 732 bp of DBR2) were labeled with alkaline phosphatase using the AlkPhos Direct Labeling Kit (Amersham Bioscience, USA). Prehybridization, hybridization (incubated at 60 °C overnight) with alkaline phosphatase labeled probe and subsequently washings of the membrane were carried out according to the AlkPhos Direct Labeling Kit protocol. Detection was performed using CDP-Star detection reagent following the manufacturer’s directions (Amersham Bioscience).

### 3.4. Reverse Transcription-Polymerase Chain Reaction Analysis

The complete transgenic lines, 1240/1, 1240/2 and 1250/1, were further analyzed by RT-PCR. Non-transformed plants as well as the incomplete T-DNA transformation line, 1250/2, were used as a negative control. The total RNA was isolated from the leaves with the QuantumPrep AquaPure RNA Isolation kit (BioRad, Hercules, CA, USA) according to the manufacturer’s recommendations. The obtained RNA was additionally purified from DNA impurities via treatment with DNAse (Thermo Fisher Scientific, Waltham, MA, USA). RNA preparations were checked for the absence of DNA contamination by PCR analyses without reverse transcription step (Figure S1B). Reverse transcription was performed using an M-MuLV RT enzyme (Thermo Fisher Scientific, USA) and oligo d(T)_16_ primer following the manufacturer’s directions. The obtained cDNA preparations were analyzed by PCR for the presence of target sequences. The primer sequences and RT-PCR conditions are presented in Table S2 of Supplementary Material.

### 3.5. Artemisinin Assay in the Transgenic Plants

Artemisinin assay was performed using TLC [34,35]. Chemicals used were of analytical grade. Anisaldehyde, *n*-hexane, trifluoroacetic acid (TFA), artemisinin, artemisinic acid were from Sigma (USA); glacial acetic acid, H_2_SO_4_ and methanol were from Panreac (USA); RP-18 _F254S_ TLC plate—Merck (Germany). Artemisinin and artemisinic acid were used as external standards. The standards were dissolved in methanol at of concentration of 1 mg/mL; the working solutions were prepared by diluting the stock solutions with methanol up to 125.0 and 62.25 ng/µL.

The leaves of transgenic and control non-transformed plants growing in the greenhouse were collected, dried in a hot air oven at 56 °C for 18 h and powdered. The samples (100 mg) were extracted overnight in *n*-hexane (10 mL), filtered, evaporated under N_2_ gas flow and dissolved in 1.0 mL of methanol. On the TLC plates the samples and standard solutions were applied manually, 10 μL and 1 μL of each, respectively. The linear ascending development was carried out on RP-18 _F254S_ TLC plate, 20 × 10 cm in a twin-trough chamber presaturated with mobile phase 0.2% TFA in water/ACN (35:65, *v*/*v*). The chromatogram was allowed to develop for 35 min to a height of 8 cm at room temperature (25 ± 2 °C) and 50% ± 5% relative humidity. After the development, TLC plates were dried in a current of air with the help of an air fan. The dried plate was dipped into freshly prepared reagent consisting of glacial acetic acid/concentrated H_2_SO_4_/anisaldehyde (50:1:0.5, *v*/*v*/*v*) followed by heating at 110 °C for 5 min to visualize the bands of artemisinin. TLC plates were visualized using imaging system Amersham Imager 600RGB (GE, USA). Artemisinin TLC analyses were performed in triplicate.

## Figures and Tables

**Table 1:** Summarized results of chrysanthemum transformation with vectors p1240 and p1250.

Stages of chrysanthemum transformation and analysis	**Vector**	
	1240	1250
Transformed explants	600	600
The explants with regenerants	20	18
Adventive shoots per one regenerating explant	1.2	1.3
Planted regenerants on the proliferation medium with kanamycin	24	23
The regenerants died on the proliferation medium with kanamycin	16	21
Kanamycin-resistant lines	8	2
NPT II -positive lines	8	2
Lines with all target genes	2	1
Transcription of all target genes	2	0

## Supplementary Materials

The following are available online at https://www.mdpi.com/2223-7747/9/4/537/s1, Figure S1: Quality check of DNA and RNA preparations for PCR and RT-PCR analysis, Table S1: PCR regimes and nucleotide sequences of used primers, Table S2: RT-PCR conditions and nucleotide sequences of used primers.
