# Supplementary material for: Agrobacterium-Mediated Transformation of Chrysanthemum with Artemisinin Biosynthesis Pathway Genes
Source: Plants (Basel). 2020 Apr 21;9(4):537. doi: 10.3390/plants9040537 (PMC7238074; doi:10.3390/plants9040537)
Supplement: Supplementary file 1 [file plants-09-00537-s001.pdf]

Supplementary Materials

Table S1. PCR regimes and nucleotide sequences of used primers.

Primer	Sequence, 5′ → 3′	Sequence Annealing	Amplified Fragment, bp	Annealing Temperatu re, °C	Extension Time, sec
ADS	F ggaagagctcagccatgtgt<br>R caggttagcaccgccagtaa	ADS	496	60	40
ADS	F ctatccttcgcaagacccttcc<br>R tgatcaatctcccgttcaaagtga	35S CaMV promoter<br>ADS	488 (ADS) 591 (mtADS)	55	40
DBR2	F tgatgcagctcacaaagaaggggc<br>R tccccttgagccacagcttgtaacc	DBR2	732	60	60
DBR2	F gctggtcaatcccattgcttttga<br>R caggatacgtgccataggttgcat	Sup promoter<br>DBR2	931	61	60
CPR	F atgcaatcaacaacttccgttaagttat<br>R ttaccatacatcacggagatatcttcc	CPR gene, full-length	2115	60	120
CPR	F gtggagactggggctctttc<br>R ctttggcatcaccgcaaaca	CPR gene, fragment	154	61	30
tHMGR	F acacctaataagtccaacatgg<br>R tgatactacgagagcggttg	tHMGR	1011	56	60
CYP71AV1	F atgaagagtatactaaaagcaatggcactc<br>R ctagaaacttggaacgagtaacaactca	CYP71AV1	1488	60	90
NPT II	F gctatgactgggcacaacagacaatc<br>R tccgagtacgtgctcgctcga	NPT II	381	60	30
virB	F ggctacatcgaagatcgtatgaatg<br>R gactatagcgatggttacgatgttgac	*A. tumefaciens* virB1	670	60	45

F- forward primer, *R*- reverse primer.

Amplification of the target fragments was performed using DreamTaq polymerase (Thermo Fisher Scientific, USA) in a manufacturer recommended buffer. The reaction mix contained genomic DNA (200 ng), forward and reverse primers (0.5 μM each) and 1.0 U DreamTaq polymerase in total volume of 25 μL. The reaction was performed under the following conditions: initial denaturation in 95 °C 5 min, denaturation was carried out by 94 °C 30 s, primer annealing in temperature described in the table, 30 s and extension at 72 °C in the extension time in the table with 32 cycles.

Table S2. RT-PCR conditions and nucleotide sequences of used primers.

Target Gene	Sequence, 5′ → 3′	Amplified Fragment, bp	Extension Time, sec
ADS	F ggaagagctcagccatgtgt<br>R caggttagcaccgccagtaa	496	40
tHMGR	F gcctataacaccaacgggca<br>R cgctgccttggttattcacg	229	60
CYP71AV1	F accctccactacccttggtt<br>R ggctccaggacacatccttc	242	90
CPR	F gtggagactggggctctttc<br>R ctttggcatcaccgcaaaca	154	120

DBR2	F aaccacgttacacggctgat<br>R ctagtgtaaccaccgcagca	136	60
Actin of *C. morifolium*	F tggacgtgacttgaccgatg<br>R cacctgaacctctcagcacc	228	60

F- forward primer, *R*- reverse primer.

First strand cDNA synthesis was performed using of M-MuLV reverse transcriptase (Thermo Fisher Scientific, USA) in a manufacturer recommended buffer. The one microgram of the total RNA and 100 pmol of oligo $d(T)_{16}$ primer were added into reaction buffer and first incubated at 42 °C 90 min.

Amplification of the target fragments was performed using DreamTaq polymerase (Thermo Fisher Scientific, USA) in a manufacturer recommended buffer. The reaction mix contained of 2 μL of the RT reaction mix, forward and reverse primers (0.5 μM each) and 1.0 U DreamTaq polymerase in total volume of 25 μL. The reaction was performed under the following conditions: initial denaturation in 95 °C 5 min, denaturation was carried out by 94 °C 30 s, primer annealing at 60 °C 30 s and extension at 72 °C in the extension time in the table with 35 cycles.

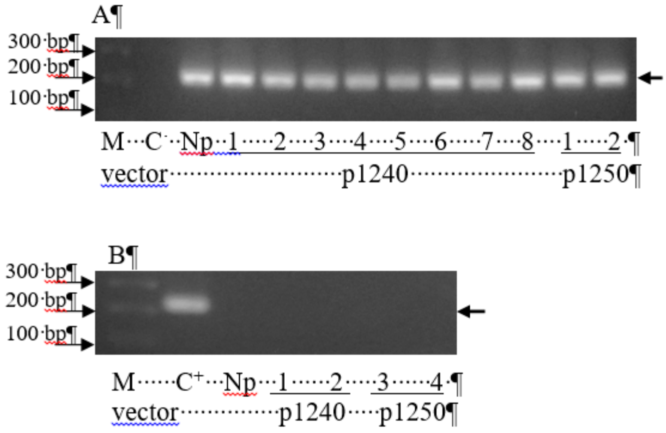

Figure S1. Quality check of DNA and RNA preparations for PCR and RT-PCR analysis. A. PCR of chrysanthemum DNA preparations using actin primers. B. PCR of chrysanthemum RNA without reverse transcription using actin primers and the expected length was 228 bp. Numbers denote independent transgenic lines, Np – non- transformed plant, C - negative control PCR reaction without adding DNA, C⁺ - chrysanthemum DNA added to RT-PCR mix, positive control. M - molecular size marker.
